# Perceived stress and depressive symptoms not neuropsychiatric symptoms predict caregiver burden in Alzheimer’s disease: a cross-sectional study

**DOI:** 10.1186/s12877-021-02136-7

**Published:** 2021-03-12

**Authors:** Manee Pinyopornpanish, Kanokporn Pinyopornpanish, Atiwat Soontornpun, Surat Tanprawate, Angkana Nadsasarn, Nahathai Wongpakaran, Tinakon Wongpakaran

**Affiliations:** 1 Department of Psychiatry, Faculty of Medicine, Chiang Mai University, 110 Intawaroros Rd. Tambon Sriphoom, Amphur Muang, Chiang Mai, 50200 Thailand; 2 Department of Family Medicine, Faculty of Medicine, Chiang Mai University, Chiang Mai, Thailand; 3 Division of Neurology, Department of Internal Medicine, Faculty of Medicine, Chiang Mai University, Chiang Mai, Thailand; 4 Northern Neuroscience Center, Faculty of Medicine, Chiang Mai University, Chiang Mai, Thailand

**Keywords:** Caregiver, Burden, Alzheimer’s disease, Neuropsychiatric symptoms

## Abstract

**Background:**

Caregiver burden affects the caregiver’s health and is related to the quality of care received by patients. This study aimed to determine the extent to which caregivers feel burdened when caring for patients with Alzheimer’s Disease (AD) and to investigate the predictors for caregiving burden.

**Methods:**

A cross-sectional study was conducted. One hundred two caregivers of patients with AD at Maharaj Nakorn Chiang Mai Hospital, a tertiary care hospital, were recruited. Assessment tools included the perceived stress scale (stress), PHQ-9 (depressive symptoms), Zarit Burden Interview-12 (burden), Clinical Dementia Rating (disease severity), Neuropsychiatric Inventory Questionnaires (neuropsychiatric symptoms), and Barthel Activities Daily Living Index (dependency). The mediation analysis model was used to determine any associations.

**Results:**

A higher level of severity of neuropsychiatric symptoms (*r* = 0.37, *p* < 0.01), higher level of perceived stress (*r* = 0.57, *p* < 0.01), and higher level of depressive symptoms (*r* = 0.54, *p* < 0.01) were related to a higher level of caregiver burden. The direct effect of neuropsychiatric symptoms on caregiver burden was fully mediated by perceived stress and depressive symptoms (*r* = 0.13, *p* = 0.177), rendering an increase of 46% of variance in caregiver burden by this parallel mediation model. The significant indirect effect of neuropsychiatric symptoms by these two mediators was (*r* = 0.21, *p* = 0.001).

**Conclusion:**

Caregiver burden is associated with patients’ neuropsychiatric symptoms indirectly through the caregiver’s depressive symptoms and perception of stress. Early detection and provision of appropriate interventions and skills to manage stress and depression could be useful in reducing and preventing caregiver burden.

**Supplementary Information:**

The online version contains supplementary material available at 10.1186/s12877-021-02136-7.

## Background

Alzheimer’s Disease (AD) composes from 60 to 80% of dementia cases [1] and imposes a severe burden on patients themselves and their relatives. Caregiving for patients with AD could be related to high levels of strain. As dementia impairs the cognitive skills and functions of patients, it increases dependency and could influence the sense of burden among caregivers. This could affect the care quality received by patients, leading to complications and poor health outcomes [2]. Any intervention that could prevent or manage caregiver burden would be beneficial to the patient and family members [3].

Caregiver burden refers to the state in which one perceives physical and psychological well-being, financial status, and social relations could be threatened by care provision [4]. Related studies have reported possible predictors of caregiver burden including patient factors (e.g., changes in symptoms and severity of the psychological and behavioral problems), and caregiver factors (e.g., younger caregiver age, being female, lower educational levels), and lack of support [5–8].

Neuropsychiatric symptoms or behavioral and psychological symptoms of dementia (BPSD) have been found to create caregivers’ distress, burden, and burnout, and might be a predictive factor for caregiver decisions to institutionalize patients [9–12]. These symptoms include emotional, perceptual and behavioral disturbances [13]. Several studies have paid attention to the relationship between neuropsychiatric symptoms and the level of caregiver burden [14, 15]. However, the evidence is inconclusive as to which types of BPSD influence caregiver well-being more than others [16].

Some studies have attempted to focus on which BPSD symptom has the greatest impact on caregiver burden and found inconsistent results due to the heterogeneity in measurement and analysis method. While practically every single symptom is important and accounted for caregiver burden and lack of well-being, the magnitude of the relationship between each neuropsychiatric symptom and caregiver burden may vary. What is lacking in most studies is a determination of what variables link neuropsychiatric symptoms and caregiver burden.

**Fig. 1:**
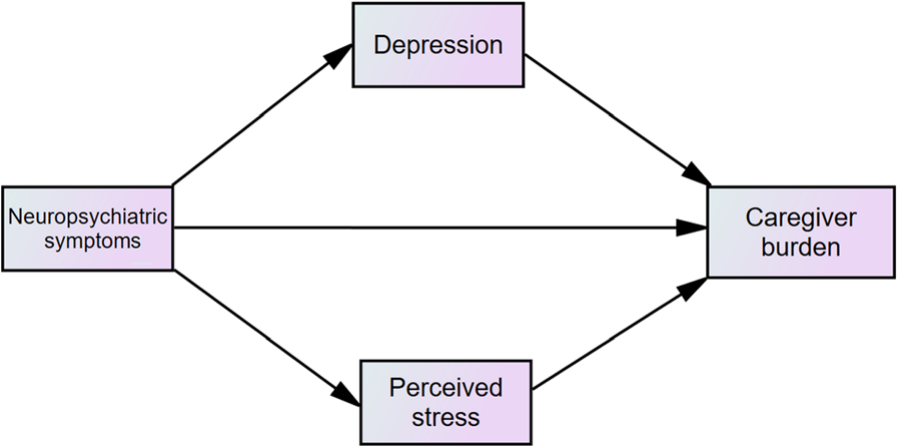
Hypothesized Model. The hypothesized parallel mediation model showing both caregiver’s perceived stress and depression mediates the relationship between neuropsychiatric symptoms and caregiver burden

Research has shown that depression is a variable involved with both neuropsychiatric symptoms and caregiving burden. Stress is also increased among caregivers [17–19]. In addition to the relationship between caregiving and depression, a significant relationship between perceived stress and depression has long been demonstrated in various settings. Evidence shows that perceived stress significantly correlated with higher scores in depressive symptoms [20–23]. It has been assumed that both perceived stress and depression would mediate the effect of neuropsychiatric symptoms on caregiver burden. To the best of our knowledge, this has not been investigated. This study aimed to investigate the associations between neuropsychiatric symptoms and caregiver burden and the mediating role of perceived stress and depressive symptoms. We hypothesized associations between neuropsychiatric symptoms and caregiver burden as described below (Fig. 1).

- Hypothesis 1: Neuropsychiatric symptoms predict caregiver burden via depressive symptoms.
- Hypothesis 2: Neuropsychiatric symptoms predict caregiver burden via perceived stress.
- Hypothesis 3: Both depressive symptoms and perceived stress mediate the relationship between neuropsychiatric symptoms in predicting caregiver burden.

## Methods

### Participants and procedure

We surveyed caregivers of patients with AD treated by neurologists at Maharaj Nakorn Chiang Mai Hospital, a tertiary care hospital. We recruited primary caregivers aged 18 years or older who had been providing care for at least 1 month. The reason why we required the caregivers to provide care for at least 1 month to participate was that this duration would provide sufficient time for caregivers to know and be exposed to the patient’s symptoms and behaviors.

**Fig. 2:**
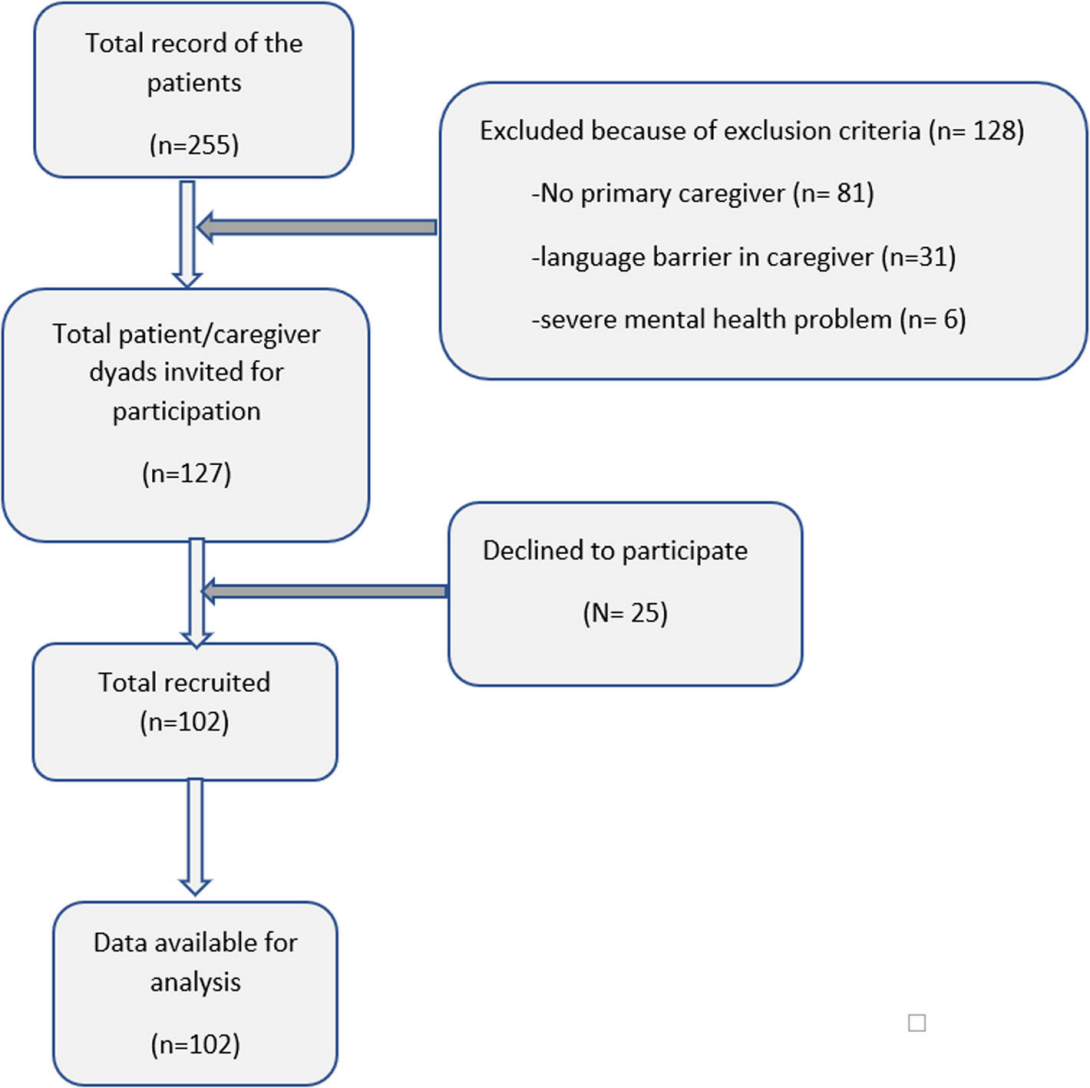
Primary caregiver flowchart

Inability to communicate, due to either language barrier or severe mental health problems, was criteria for exclusion. There were 127 patient/caregiver dyads invited to participate and 102 dyads were included for final analysis (Fig. 2). Caregivers were interviewed using a structured questionnaire to evaluate the patient’s status and to collect data including age, sex, occupation, highest level of education, relationship with patient and duration of care. The caregiver’s perception of the patients’ disease severity and functional performance were evaluated using Clinical Dementia Rating (CDR), Neuropsychiatric Inventory Questionnaires (NPI-Q) and Barthel Activities Daily Living Index (ADL). The caregiver’s stress, depressive symptoms, quality of life and feeling of burden were determined using the Thai version of the perceived stress scale (PSS), Patient Health Questionnaire-9 (PHQ-9), and the Zarit Burden Interview (ZBI-12). The caregivers provided written informed consent before completing the questionnaires, and provided consent on the patients’ behalf.

### Measures

#### Measures about patients’ status

##### Clinical dementia rating (CDR)

CDR is a 5-point scale used to characterize 6 domains of cognitive and functional performance applicable to AD [24]. This includes memory, orientation, judgment and problem-solving ability, community affairs of the subject, home life, hobbies and personal care. It is based on a scale of 0, 0.5, 1, 2, 3 (from no cognitive impairment to severe cognitive impairment). The scores for each of the categories are weighted equally and summed to obtain a total score (0 to 18). Higher scores indicate greater severity of dementia. Translations of the CDR have been shown valid and reliable in Asian populations.

##### Neuropsychiatric inventory questionnaires (NPI-Q)

A structured interview of the caregiver is used to assess 12 behavior domains during the past month by asking whether the symptom is present (1 = yes, 0 = no) and how severe the symptom is (1 = mild, 2 = moderate, 3 = severe). The 12 domains include delusions, hallucinations, dysphoria, euphoria, anxiety, agitation, apathy, irritability, disinhibition, aberrant motor behavior, sleep and appetite/eating disorder [25].

##### Barthel activities daily living index (ADL)

The ADL is scored as 0 to 1, 2, or 3 in each of 10 basic domains including feeding, hygiene, transfer, toileting, ambulation, dressing, stairs, bathing and continence (stool and urine) [26]. Higher scores of ADL indicate better daily function.

#### Measures about caregivers

##### The Thai version of the 10-item perceived stress scale (PSS or T-PSS-10)

The PSS is a self-reporting, 10-item questionnaire measuring the extent to which individuals perceived stress [27]. The 5-response Likert scale ranges from 0 (never) to 4 (very often); the total scores range from 0 to 40. Higher scores indicate higher levels of perceived stress. This Thai version also shows good psychometric properties. The 2-factor structure model, namely, stress and control, fit well with the data in the confirmatory factor analysis study [28].

##### Patient health Questionnaire-9 (PHQ-9)

The PHQ-9 is a self-reporting, 9-item questionnaire measuring the extent to which an individual experienced depressive symptoms over the past 2 weeks [29]. The 4-response Likert scale ranges from 0 (not at all) to 3 (nearly every day); the total scores range from 0 to 27 and higher scores indicate higher levels of depressive symptoms. The Thai version showed good psychometric properties [30].

##### The 12-item Zarit burden interview (ZBI-12)

The ZBI is a 12-item caregiver-reported questionnaire measuring the burden the respondent feels in providing care to the patient. Higher scores indicate a higher burden. The total score equals 17 [31]. This shortened, Thai version of the burden scale shows the ability to detect caregiver burden as a global score and is related to the 22-item version [32].

#### Data analysis

Descriptive analyses, such as sociodemographic data and all clinical variables, were presented as frequency, percentage and mean standard deviation. For mediation model analysis, we first conducted correlation analyses to assess the associations between all variables. Spearman’s rank correlation or Pearson’s correlation analysis were utilized as appropriate. To meet the assumption of the mediation model [33], a significant correlation (*p* < .05) between independent variables (NPI -Q score) and mediators (PHQ-9 score and PSS score), and dependent variables (ZBI score) would be expected. We then conducted a mediation model to test Hypothesis 1: that neuropsychiatric symptoms predict caregiver burden via depressive symptoms (Model 2). In Model 3, perceived stress was specified to mediate the relationship between neuropsychiatric symptoms and caregiver burden, and in Model 4, depressive symptoms and perceived stress were specified as parallel mediators of the relationship between neuropsychiatric symptoms and caregiver burden. Despite the fact that a serial mediation model (M1 = perceived stress; M2 = depressive symptoms) could be an alternative as, theoretically, stress precedes depression, the sample size did not allow us to perform such a complex model (see supplement for power calculation). All models were conducted using Mplus, Version 8.0 with Maximum Likelihood estimation. To test the model fitness, a nonsignificant χ2 test; an RMSEA < 0.06; an SRMR < 0.08; a comparative fit index (CFI) and a Tucker–Lewis Index (TLI) ≥ 0.95 [34], were considered a good model fit. Indirect effects were tested using bootstrap estimation with 5000 samples. Bias-corrected percentile bootstrap confidence intervals were reported at the 95% confidence level. All diagrams were drawn using AMOS version 18.

## Results

One hundred two patients together with their caregivers were recruited in the study including 72 female patients (70.6%) with a mean age of 79.4 years (SD 7.9). The average ADL score was 15.4 (SD 6.5) and all patients had at least one symptom of BPSD (mean 5.8, SD 2.6). The average age of caregivers was 55.0 (SD 12.9). Most caregivers in our sample group were educated, female (77.5%), and without financial problems. About half reported physical problems. Most caregivers were a child of the patient (68.6%). The average duration of care of the patient was 6.1 years (SD 8.7), which indicated the presence of outliers. Concerning the outliers, we have analyzed the correlation between ZBI and duration of caregiving both with original data and data without outliers, and the results were shown to be the same - without significance. The average time spent taking care of the patient was 15.4 h daily (SD 8.1) and most patients had at least one helper. About 16% reported having severe burden. The demographic data of the patients and caregivers are described in Table 1**.**

**Table 1:** Demographic Data of the Patients and Caregivers

**Patients’ Characteristics (*N*** **= 102)**	
Female sex, n(%)	72 (70.6)
Age, mean ± SD	79.4 ± 7.9
Years of education, mead ± SD	7.1 ± 4.9
Marital status	
• Married	44 (43.1)
• Widow/widower	51 (50.0)
• Single	5 (4.9)
• Divorced/separated	2 (2.0)
Duration of disease (years), mean ± SD	2.53 ± 3.1
AD type	
• AD	63 (61.8)
• AD+CVD	39 (38.2)
Severity of disease by CDR, mean ± SD	9.74 (4.65)
ADL, mean ± SD	15.4 ± 6.5
Neuropsychiatric symptoms	
• Number of symptoms, mean ± SD	5.8 (2.6)
• NPI score, mean ± SD	20.3 (21.5)
**Caregivers’ Characteristics (*N*** **= 102)**	
Female sex, n (%)	79 (77.5)
Age (years), mean ± SD	55.0 ± 12.9
Years of education, mean ± SD	13.8 (4.6)
Relationship with patient, n (%)	
• Spouse	21 (20.6)
• Parent	1 (1.0)
• Child	70 (68.6)
• Other relatives	5 (4.9)
• Hired caregiver	5 (4.9)
Financial problem	
• No	81 (79.4)
• Some problem without debt	21 (7.8)
• Some debt	4 (3.9)
• Lots of debt	9 (8.8)
Having a health problem	
• Physical health problem	53 (52.0)
• Mental health problem	8 (7.8)
Duration of care (years), mean ± SD	6.1 ± 8.7
Hour of care per day, mean ± SD	15.4 ± 8.1
Having at least one helper, n(%)	83 (82.4)
PSS, mean ± SD	14.6 ± 4.9
PHQ-9, mean ± SD	4.1 ± 3.9
• mild depression (7–12)	20 (19.5)
• moderate depression (13–18)	1 (1.0)
• severe depression (≥19)	2 (2.0)
ZBI-12, mean ± SD	10.6 (7.5)
• Severe/high burden (≥17)	16 (15.7)

*AD* Alzheimer’s disease, *ADL* Activities Daily Living, *CDR* Clinical Dementia Rating, *CVD* Cardiovascular disease, *NPI* Neuropsychiatric Inventory Questionnaires, *PHQ-9* Patient Health Questionnaire-9, *PSS* Perceived Stress Scale, *ZBI* Zarit Burden Interview

Table 2 shows that no sociodemographic variables, CDR and ADL were significantly related to ZBI, but to PSS, PHQ-9 and NPI. Significant correlations were observed between PHQ-9, ZBI, PSS, and NPI, denoting that the mediation model among these 4 variables was justified.

**Table 2:** Zero-Order Correlation Between Variables

	Patient			Caregiver					
	CDR	ADL	NPI	Sex	Age	Education	PHQ-9	PSS	ZBI-12
CDR	1								
ADL	−0.799^**^	1							
NPI-Q	0.186	−0.142	1						
Sex	0.088	−0.063	− 0.232^*^	1					
Age	0.215^*^	−0.268^**^	− 0.021	0.069	1				
Education	− 0.029	0.004	0.023	0.005	−0.279^**^	1			
PHQ-9	0.242^*^	−0.310^**^	0.254^**^	0.146	0.006	0.141	1		
PSS	0.113	−0.121	0.311^**^	0.029	−0.208^*^	0.233^*^	0.490^**^	1	
ZBI-12	0.163	−0.187	0.370^**^	−0.098	0.002	0.193	0.540^**^	0.569^**^	1

*ADL* Activities Daily Living (to assess patient’s functional status), *CDR* Clinical Dementia Rating (to assess patient’s disease severity), *NPI* Neuropsychiatric Inventory Questionnaires (to assess patient’s neuropsychiatric symptoms), *PHQ-9* Patient Health Questionnaire-9 (to assess caregivers’ depressive symptoms), *PSS* Perceived Stress Scale (to assess perceived stress of caregiver), *ZBI* Zarit Burden Interview (to assess caregiver burden)

*p < 0.05, **p < 0.01

Table 3 shows the results of the mediation model after accounting for sex, age, education, CDR and ADL. Model 0 was used to test the association between NPI and ZBI. The model fit statistics suggested that the model fit the data, χ^2^ = 28.354, df = 11, *p* = 0.0029, CF =1.000, TLI = 1.000, RMSEA = 0.000, SRMR = 0.000. NPI explained 17.8% of caregiver burden. Model 2 tested hypothesis 1 in that PHQ-9 was added to the model. The model fit statistics suggested that the model fit the data, χ^2^ = 76.372, df = 18, *p* < 0.001; CFI =1.000, TLI =1.000, RMSEA = 0.000, SRMR = 0.000. The overall model explained 36.6% of the variance in caregiver burden. Model 3, tested hypothesis 2 in that PSS was added to the model. The model fit statistics suggested that the model fit the data, χ^2^ = 76.947, df = 18, *p* < 0.001; CFI =1.000, TLI = 1.000, RMSEA = 0.000, SRMR = 0.000. Compared with Model 1, the direct effect of NPI was reduced to 0.179, and was nonsignificant. The overall model explained 39.3% of the variance in caregiver burden. Model 4, tested hypothesis 3 in that both PSS and PHQ-9 were added to the parallel model. The model fit statistics suggested that the model fit the data, χ^2^ = 129.408, df = 26, *p* < 0.001; CFI = 1.000, TLI = 1.000, RMSEA = 0.000, SRMR = 0.000. Notably, the effect of PSS was stronger than that of PHQ-9; both nullified the direct effect of NPI. The overall model explained 45.9% of the variance in caregiver burden.

**Table 3:** Mediation Effect of Perceived Stress and Depression on the Relationship Between Neuropsychiatric Symptoms on Caregiver Burden Scores^a^

	Outcome: ZBI-12		Product of coefficients		*p*-value	Bootstrapping Bias-corrected 95%CI	
			Point estimate	SE		Lower limit	Upper limit
Model 1 (*R*^2^ = 0.178)	Predictor: NPI	Mediator: -					
	Total/direct effect		0.339	0.109	0.002	0.150	0.513
Model 2 (*R*^2^ = 0.366)	Predictor: NPI	Mediator: PHQ-9					
	Total effect		0.339	0.119	0.002	0.148	0.513
	*Direct effect*						
	- NPI		0.206	0.101	0.040	0.041	0.366
	*Indirect effect*						
	-via PHQ-9		0.133	0.049	0.006	0.082	0.293
Model 3 (*R*^2^ = 0.393)	Predictor: NPI	Mediator: PSS					
	Total effect		0.339	0.109	0.002	0.205	0.595
	*Direct effect*						
	- NPI		0.179	0.110	0.104	−0.004	0.355
	*Indirect effect*						
	-via PSS		0.160	0.058	0.006	0.086	0.351
Model 4 (*R*^2^ = 0.459)	Predictor: NPI	Mediator: PSS/PHQ-9					
	Total effect		0.340	0.109	0.002	0.151	0.513
	*Direct effect*						
	-NPI		0.134	0.099	0.177	−0.030	0.293
	*Indirect*		0.206	0.061	0.001	0.119	0.318
	- via PSS		0.117	0.049	0.016	0.050	0.214
	- via PHQ-9		0.089	0.042	0.036	0.033	0.185

*SE* Standard Error, *CI* Confidence Interval, *NPI* Neuropsychiatric Inventory Questionnaires, *PHQ-9* Patient Health Questionnaire-9, *PSS* Perceived Stress Scale, *ZBI* Zarit Burden Interview

^a^Covariates were accounted for, i.e. Age, sex, years of education, level of clinical dementia rating, and level of activity of daily life

## Discussion

The present study aimed to demonstrate to what extent caregiver burden was influenced by neuropsychiatric symptoms. The patient’s neuropsychiatric behavioral symptoms were associated with the feeling of burden reported by the caregiver. This result is concurrent with many studies [9, 15, 35–37]. However, by adding depressive symptoms and perceived stress, the feeling of burden could be more fully explained while reducing the direct effect of neuropsychiatric symptoms to nonsignificance.

This indicated that a feeling of burden comes from many sources. Whatever the source is, it entails experiencing stress and depressive symptoms in the caregiver. The fact that depressive symptoms and perceived stress fully mediated the relationship with the severity of neuropsychiatric symptoms may indicate that depressive symptoms and perceived stress are more proximal to caregiver burden than the patient’s symptoms. By including depressive symptoms and perceived stress in the parallel mediation model, the variance of caregiver burden was explained up to 28%. These findings were consistent with related studies that show the caregiver’s factor plays an important role, especially concerning depression [38, 39].

Our findings were also supported by Wang and colleagues, in that depression had a significantly direct effect on caregiver burden. They also found that the relationship could be moderated by perceived social support [40], while other research found it could be mediated by a positive attitude concerning caregiving [41].

Notably, those findings, including ours, have been observed in Asian samples, which might differ from studies conducted in a Western culture, where caregiving activity may not be provided mostly by family members or relatives. As noted by Yu et al., in Chinese culture (comparable to Thai culture), caring for parents is considered the role and responsibility of family members. In addition to social support, they found that family function and caregiving experience were mediators of the relationship between the patient’s factors, e.g., cognitive level, physical function behavioral problems and caregiver burden [42]. However, similar findings were found in non-Asian cultures as well, in that the caregiver’s age and type of relationship showed a higher impact on the neuropsychiatric symptoms and caregiver burden [38, 39].

Does the effect size of perceived stress over depression on caregiver burden matter clinically? We have known that perceived stress could lead to depression, and vice versa [43–45]. Perceived stress is considered a predetermined psychological state before experiencing depression. Perceived stress should then become a target for early identification in managing caregiving problems. As mentioned earlier, the serial mediation model, in which perceived stress has a direct effect on depressive symptoms, should be proposed and tested if the sample size is sufficient. Despite the fact that the sample size was relatively small, it exhibited sufficient statistical power (≥80%) for each parameter. However, it did not support the case for the serial mediation model, which requires up to 280 participants (see supplement file).

Interestingly, our results showed that perceived stress was related to caregiver’s education and age. Individuals with a younger age, but a higher level of education, tend to feel more stressed in providing care to patients with AD, while depression was associated with the patient’s symptoms, such as the severity of the dementia and the ability to help oneself. These interesting relationships are not fully clear to us and need further investigation. However, the fact that perceived stress overshadowed depression and makes patient’s factors seem less significant could be explained by its proximity between perceived stress and burden, and variances shared by the same respondent.

Therefore, to prevent caregiver burden, identifying and managing these two mental health problems could prove useful. While physicians assess the severity and progression of the disease, the perception of the caregiver should be simultaneously assessed. When the caregiver feels stress, difficulty in controlling neuropsychiatric symptoms of the patient, or other problems, then caregiver training could be useful. Studies also revealed an association in the opposite direction: caregiver burden might affect patient behaviors. Inappropriate caregiver management strategies, such as confronting or ignoring the patient, or poor patient-caregiver interaction may trigger dementia in patients’ behaviors [46]. Effective treatment could include both pharmacologic and non-pharmacologic strategies. Nonpharmacological interventions to reduce patient’s neuropsychiatric symptoms include music therapy, touch therapy, bright light therapy, cognitive rehabilitation, etc. However, indications for each intervention could vary depending on the type of neuropsychiatric symptom. Caregiver training, especially among younger people, should include educating and supporting the caregiver to handle those symptoms and use appropriate coping skills. A recent study of systematic review and meta-analysis showed that interventions including emotional support, exercise, and education/skills-based training on coping strategies and dealing with patient behaviors, trended to relieve caregiver burden. This was especially evident when the interventions consisted of multicomponent content. These interventions would reduce stress or depression, and ultimately reduce the feeling of burden [47–50].

The strengths of the present study are that it focused on patients with AD and their primary caregivers. Caregivers who participated in the study were selected as a key primary caregiver who would be most affected by the patient. However, some limitations were encountered. Firstly, the study recruited participants from a tertiary care hospital which would not represent caregivers and patients with AD being treated elsewhere. Secondly, according to the nature of the cross-sectional study design, a causal relationship could not be evaluated. Thirdly, a caregiver’s depressed mood or presenting mental disorder during the interview could have led to information bias, as any abnormal judgment stemming from the mental disorder would interfere with the analysis. This group would be associated with more feelings of burden, so this could have resulted in an underestimated prevalence. Thus, initially in the analysis plan, we have endeavored to minimize this effect by excluding caregivers with a severe mental disorder from the study. However, such cases were not found during the recruitment so this would not have affected the results. Fourthly, family function is important, especially in Asian culture, and may have an impact on the feelings of burden. Unfortunately, we did not collect such specific information. Lastly, the small sample size precludes us from testing a more specific model determining the direct effect of perceived stress on depressive symptoms, i.e. a serial mediation model. In conclusion, caregiver burden could not be neglected when taking care of patients with AD. Caregiver’s feeling of burden concerning the patient’s neuropsychiatric symptoms is indirectly associated with the caregiver’s perception of stress and feeling of depression. Apart from controlling patients’ symptoms, early detection and providing psychological support, effective intervention and appropriate emotional management to the caregivers would provide benefit in reducing and preventing caregiver burden.

## Supplementary Information

**Additional file 1.**

## Data Availability

The datasets used and/or analyzed during the current study are available from the corresponding author on reasonable request.

## Abbreviations

AD: Alzheimer’s Disease
ADL: Activities Daily Living
CDR: Clinical Dementia Rating
NPI: Neuropsychiatric Inventory Questionnaires
PHQ-9: Patient Health Questionnaire-9
PSS: Perceived Stress Scale
ZBI: Zarit Burden Interview
