# Supplementary material for: Perceived stress and depressive symptoms not neuropsychiatric symptoms predict caregiver burden in Alzheimer’s disease: a cross-sectional study
Source: BMC Geriatr. 2021 Mar 12;21:180. doi: 10.1186/s12877-021-02136-7 (PMC7953798; doi:10.1186/s12877-021-02136-7)
Supplement: Supplementary file 1 — Additional file 1. [file 12877_2021_2136_MOESM1_ESM.docx]

Supplement

Supplementary File:

Sample size and Statistical power based on Schoemann, et al.’s Monte Carlo Method (Schoemann, Boulton, & Short, 2017)

To generate data (power and sample size) for both mediation models, we entered standardized coefficients that allow the application to compute a covariance matrix for all variables in the model.

| Model | N | Number of Replications | Monte Carlo Draws per Rep | Random Seed | Confidence Level (%) | Parameter | Power (95%CI) | No. of sample size needed to reach power of 80% |
| --- | --- | --- | --- | --- | --- | --- | --- | --- |
| Parallel  mediation | 102 | 1000 | 20000 | 1234 | 95 | a1b1 | 0.91(0.88-0.94) | - |
|  | 102 | 1000 | 20000 | 1234 | 95 | a2b2 | 0.80(0.76-0.83) | - |
|  |  |  |  |  |  |  |  |  |
|  |  |  |  |  |  |  |  |  |
| Serial  mediation | 102 | 1000 | 20000 | 1234 | 95 | a1b1 | 0.50(0.45-0.54) | 164 |
|  | 102 | 1000 | 20000 | 1234 | 95 | a2b2 | 0.36(0.32-0.40) | **280** |
|  |  |  |  |  |  | a1db2 | 0.88(0.84-0.91) | - |

| Parallel mediation model | Serial mediation model |
| --- | --- |
| 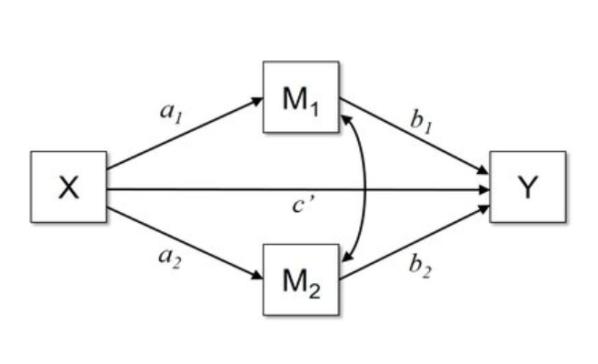 | 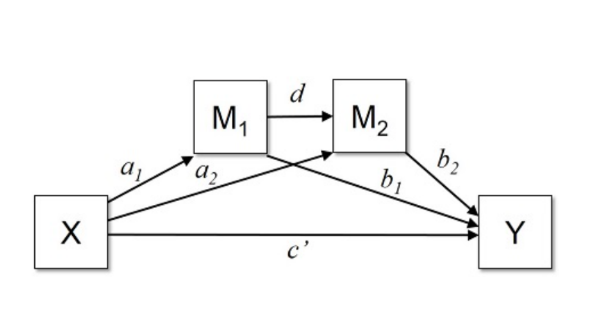 |

Reference:

Schoemann, A. M., Boulton, A. J., & Short, S. D. (2017). Determining Power and Sample Size for Simple and Complex Mediation Models. *Social Psychological and Personality Science, 8*(4), 379-386. doi:10.1177/1948550617715068
